# rs1760944 Polymorphism in the APE1 Region is Associated with Risk and Prognosis of Osteosarcoma in the Chinese Han Population

**DOI:** 10.1038/s41598-017-09750-9

**Published:** 2017-08-24

**Authors:** Xing Xiao, Yun Yang, Yanjun Ren, Debo Zou, Kaining Zhang, Yingguang Wu

**Affiliations:** 1 Department of Spine Surgery, Shandong Provincial Qianfoshan Hospital, Jinan, Shandong China; 2 School of Medicine, Shandong University, Jinan, Shandong China

## Abstract

The effects of single nucleotide polymorphisms (SNPs) at APE1 have been investigated in several types of cancer. However, no reports of the association of APE1 polymorphisms with osteosarcoma (OS) have been published. The present study was designed to determine whether APE1 polymorphisms (rs1130409, rs1760944, rs1760941, rs2275008, rs17111750) are associated with OS. A 2-stage case-control study was performed in a total of 378 OS patients and 616 normal controls. Individuals carrying TG and GG genotypes had significantly lower risk of developing OS than those with the WT genotype TT at rs1760944 (OR = 0.65, 95%CI 0.49–0.86; OR = 0.50, 95%CI 0.34–0.74, respectively). OS patients with allele G at rs1760944 were less susceptible to low differentiation tumor and metastasis (OR = 0.73, 95%CI 0.54–0.98; OR = 0.63, 95%CI 0.43–0.92, respectively). Kaplan-Meier curves and log-rank results revealed that OS patients harboring genotype GG and G allele at rs1760944 had better survival (*P* < 0.001 for both). In addition, the APE1 protein was underexpressed in individuals who carried G allele at rs1760944. This study suggested that APE1 rs1760944 polymorphism is associated with decreased risk of developing OS and better survival of OS patients.

## Introduction

Osteosarcoma (OS) is an aggressive malignant bone cancer that mainly affects children, adolescents, and young adults, comprising approximately 20% of all bone tumors and 5% of pediatric tumors overall^1^. Current OS treatment mainly involves standard chemotherapy administered before and after surgery, followed by radiation. This regimen has a 5-year survival rate of 60–70% in OS patients^2^. However, the survival rate of OS patients with locally advanced or metastatic tumor upon diagnosis or recurrence was still low, and the median survival time for these patients was found to be around 23 months^3^. Although the development of chemotherapy and targeted agents may improve the response to treatment in a subset of OS patients, but it did not significantly increase overall survival^2, 4^. Therefore, a global understanding of the underlying factors affecting OS biology could assist in the identification of diagnostic and prognostic biomarkers and therapeutic targets for the management of OS patients.

DNA damage repair refers to a cellular response that restores the normal nucleotide sequence and stereochemistry of DNA following DNA damage^5^. Base excision repair (BER) is one part of the DNA repair system, which remediates DNA damage caused by oxidative reagents and alkylation and contributes to the integrity and stability of the genome and defense from mutations^6^. DNA damage could be reversed and removed by base excision repair mechanisms to protect humans from carcinogenesis^7^. The apurinic/apyrimidinic endonuclease (*APE1*) gene is located on chromosome 14q11.2-q12 from 20,455,131 bp to 20,457,772 bp, which encodes an enzyme belonging to the BER pathway. As one of the key genes in the BER pathway, APE1 identifies and splits phosphodiester bonds via a hydrolytic mechanism on the 5′ side of abasic sites, thus specifically activating DNA repair^8^. APE1 can also participate in other cellular processes such as the response to oxidative stress, cell cycle control, and apoptosis^9^. In recent years, many studies have reported that single-nucleotide polymorphisms (SNPs) in the *APE1* gene are associated with the risk of specific cancers such as lung cancer, cervical cancer, ovarian cancer, and prostate cancer^10–13^.

OS is a multi-factorial disease. Many factors are known to play an essential role in the development of OS, including environmental and genetic factor^14^. Hereditary factors have also been found to be significantly associated with OS, particularly regarding gene polymorphisms in the DNA repair systems such as *ERCC*, *XPD*, and *GST* ^15–17^. However, until now, there has been a scarcity of data regarding the association of APE1 polymorphism with OS patients. Therefore, to determine whether variation in APE1 could modify the risk of OS, a case–control study was conducted to evaluate the association between APE1 polymorphisms and the risk of OS in 2 cohorts of Chinese Han individuals.

## Results

### Participant characteristics

Clinical and demographic information of the enrolled OS cases and control were described in Table 1. No significant differences could be observed between OS cases and controls for both cohorts in terms of age and sex distribution (both *P* > 0.05).

**Table 1:** Clinical characteristics of OS patients and normal controls.

Characteristic	Cohort 1		P	Cohort 2		P
	OS Cases (n = 172)	Controls (n = 256)		OS Cases (n = 206)	Controls (n = 360)	
Age (Years)						
≤20	97	151	0.62	116	201	0.93
>20	75	105		90	159	
Gender (n, %)						
Male	99	162	0.68	114	193	0.73
Female	63	94		92	167	
Tumor Location						
Femur	93			104		
Tibia/Fibula	49			61		
Other	30			41		
Tumor Size (cm)						
< 6	90			104		
≥ 6	82			102		
Tumor Grade						
High	85			108		
Low	87			98		
Metastasis at Diagnosis						
Negative	137			159		
Positive	35			47		
Response to Chemotherapy						
Good	92			107		
Poor	80			99		

### Association of 5 APE1 polymorphisms with risk of OS in cohort 1

In cohort 1, all 5 APE polymorphisms were genotyped in 172 OS patients and in 256 normal controls. In normal controls, none of the genotype frequencies of the 5 selected SNPs deviated from the Hardy-Weinberg equilibrium (all *P* > 0.10). As shown in Table 2, significant differences between OS patients and healthy controls were observed in rs1760944. The frequencies of the TG and GG genotypes were significantly decreased and individuals carrying TG and GG genotypes showed a significant decrease in the risk of development of OS (OR = 0.62, 95%CI 0.41–0.95; OR = 0.48, 95%CI 0.27–0.87; respectively). Significantly decreased risk for OS was also observed in subjects with TG and GG genotype under a recessive model (OR = 0.58, 95%CI 0.39–0.87). Similarly, compared with T allele, individuals carrying G allele had significantly lower risk of developing OS (OR = 0.66, 95%CI 0.50–0.88). In addition, no significant differences in the other 4 polymorphisms were detected between OS patients and healthy controls.

**Table 2:** Genotype frequencies of APE1 polymorphisms among OS patients and normal control.

	Cohorts	Genotype	OS Cases	Controls	HWE	OR (95%CI)	P
rs1130409	Cohort 1	TT	64 (37.2)	90 (35.1)	0.32	Reference	
		TG	79 (45.9)	117 (45.7)		0.95 (0.62–1.46)	0.82
		GG	29 (16.9)	49 (19.2)		0.83 (0.48–1.46)	0.57
		TG + GG	108 (62.8)	166 (64.9)		0.91 (0.61–1.37)	0.68
		T	207 (60.2)	297 (58.0)		Reference	
		G	137 (39.8)	215 (42.0)		0.94 (0.69–1.21)	0.57
	Cohort 2	TT	70 (34.0)	118 (32.8)	0.45	Reference	
		TG	99 (48.1)	170 (47.2)		0.98 (0.67–1.44)	0.51
		GG	37 (17.9)	72 (20.0)		0.87 (0.53–1.42)	0.62
		TG + GG	136 (66.0)	242 (67.2)		0.95 (0.66–1.36)	0.78
		T	239 (58.0)	406 (56.4)		Reference	
		G	173 (42.0)	314 (43.6)		0.94 (0.73–1.20)	0.62
	Combined	TT	134 (34.4)	208 (33.8)		Reference	
		TG	178 (47.1)	287 (46.6)		0.96 (0.72–1.28)	0.83
		GG	66 (17.5)	121 (19.6)		0.85 (0.59–1.23)	0.40
		TG + GG	244 (64.6)	408 (66.2)		0.93 (0.71–1.22)	0.63
		T	446 (59.0)	703 (57.1)		Reference	
		G	310 (41.0)	529 (42.9)		0.92 (0.77–1.11)	0.40
rs1760944	Cohort 1	TT	80 (46.5)	86 (33.6)	0.58	Reference	
		TG	70 (40.7)	121 (47.3)		**0.62 (0.41–0.95)**	**0.032**
		GG	22 (12.8)	49 (19.1)		**0.48 (0.27–0.87)**	**0.015**
		TG + GG	92 (53.5)	170 (76.4)		**0.58 (0.39–0.87)**	**0.009**
		T	230 (66.9)	293 (57.2)		Reference	
		G	114 (33.1)	219 (42.8)		**0.66 (0.50–0.88)**	**0.005**
	Cohort 2	TT	83 (40.3)	108 (30.0)	0.97	Reference	
		TG	93 (45.1)	178 (49.4)		**0.68 (0.47–0.99)**	**0.04**
		GG	30 (14.6)	74 (20.6)		**0.53 (0.32–0.88)**	**0.017**
		TG + GG	123 (59.7)	252 (70.0)		**0.64 (0.44–0.91)**	**0.016**
		T	259 (62.8)	394 (54.7)		Reference	
		G	153 (37.2)	326 (45.3)		**0.71 (0.56–0.92)**	**0.009**
	Combined	TT	163 (0.43)	194 (0.31)		Reference	
		TG	163 (0.43)	299 (0.49)		**0.65 (0.49–0.86)**	**0.003**
		GG	52 (0.16)	123 (0.20)		**0.50 (0.34–0.74)**	**<0.01**
		TG + GG	215 (0.59)	422 (0.69)		**0.61 (0.47–0.79)**	**<0.01**
		T	489 (0.65)	687 (0.56)		Reference	
		G	267 (0.35)	545 (0.44)		**0.69 (0.57–0.83)**	**<0.01**
rs2275008	Cohort 1	TT	125 (0.73)	200 (0.78)	0.187	Reference	
		TC	43 (0.25)	50 (0.20)		1.38 (0.86–2.19)	0.18
		CC	4 (0.02)	6 (0.02)		1.07 (0.30–3.86)	0.58
		TC + CC	47 (0.27)	56 (0.22)		1.34 (0.86–2.10)	0.21
		T	293 (0.85)	450 (0.88)		Reference	
		C	51 (0.15)	62 (0.12)		1.26 (0.85–1.88)	0.26
	Cohort 2	TT	151 (0.73)	273 (0.76)		Reference	
		TC	51 (0.25)	78 (0.21)		1.18 (0.79–1.77)	0.47
		CC	4 (0.02)	9 (0.03)		0.80 (0.24–2.65)	0.49
		TC + CC	55 (0.27)	87 (0.24)		1.14 (0.77–1.69)	0.55
		T	353 (0.86)	624 (0.87)		Reference	
		C	59 (0.14)	96 (0.13)		1.08 (0.77–1.54)	0.65
	Combined	TT	276 (0.73)	473 (0.77)	0.08	Reference	
		TC	94 (0.25)	128 (0.21)		1.26 (0.93–1.71)	0.16
		CC	8 (0.02)	15 (0.02)		0.91 (0.38–2.18)	0.51
		TC + CC	102 (0.27)	143 (0.23)		1.22 (0.91–1.64)	0.20
		T	646 (0.85)	1074 (0.87)		Reference	
		C	110 (0.15)	158 (0.13)		1.16 (0.89–1.50)	0.28
rs17111750	Cohort 1	CC	105 (0.61)	156 (0.61)	0.121	Reference	
		CT	49 (0.28)	82 (0.32)		0.89 (0.58–1.37)	0.66
		TT	18 (0.11)	18 (0.07)		1.49 (0.74–2.99)	0.28
		CT + TT	67 (0.39)	100 (0.39)		0.99 (0.67–1.48)	0.53
		C	259 (0.75)	394 (0.77)		Reference	
		T	85 (0.25)	118 (0.23)		1.10 (0.80–1.51)	0.62
	Cohort 2	CC	125 (0.61)	183 (0.60)	0.08	Reference	
		CT	62 (0.30)	100 (0.32)		0.91 (0.62–1.34)	0.69
		TT	19 (0.09)	23 (0.08)		1.21 (0.63–2.31)	0.62
		CT + TT	81 (0.39)	123 (0.40)		0.96 (0.67–1.38)	0.85
		C	312 (0.76)	466 (0.76)		Reference	
		T	100 (0.24)	146 (0.24)		1.02 (0.76–1.37)	0.88
	Combined	CC	230 (0.61)	339 (0.60)		Reference	
		CT	111 (0.29)	182 (0.32)		0.90 (0.67–1.20)	0.51
		TT	37 (0.10)	41 (0.07)		1.33 (0.83–2.14)	0.27
		CT + TT	148 (0.39)	223 (0.40)		0.98 (0.75–1.28)	0.89
		C	571 (0.76)	860 (0.77)		Reference	
		T	185 (0.24)	264 (0.23)		1.06 (0.85–1.31)	0.66
rs1760941	Cohort 1	CC	96 (0.56)	131 (0.51)	0.184	Reference	
		CA	63 (0.37)	98 (0.38)		0.88 (0.58–1.32)	0.60
		AA	13 (0.07)	27 (0.11)		0.66 (0.32–1.34)	0.30
		CA + AA	76 (0.44)	125 (0.49)		0.83 (0.56–1.22)	0.38
		C	255 (0.74)	360 (0.70)		Reference	
		A	89 (0.26)	152 (0.30)		0.83 (0.61–1.12)	0.25
	Cohort 2	CC	117 (0.57)	194 (0.54)	0.102	Reference	
		CA	71 (0.34)	132 (0.37)		0.89 (0.62–1.29)	0.58
		AA	18 (0.09)	34 (0.09)		0.88 (0.47–1.63)	0.76
		CA + AA	89 (0.43)	166 (0.46)		0.89 (0.63–1.26)	0.54
		C	305 (0.74)	520 (0.72)		Reference	
		A	107 (0.26)	200 (0.28)		0.91 (0.69–1.20)	0.53
	Combined	CC	213 (0.56)	325 (0.53)		Reference	
		CA	134 (0.35)	230 (0.37)		0.89 (0.68–1.17)	0.40
		AA	31 (0.09)	61 (0.10)		0.78 (0.49–1.24)	0.30
		CA + AA	165 (0.44)	291 (0.47)		0.87 (0.67–1.20)	0.29
		C	560 (0.74)	880 (0.71)		Reference	
		A	196 (0.26)	352 (0.29)		0.87 (0.71–1.07)	0.22

### Association of 5 APE1 polymorphisms with risk of OS in cohort 2

To confirm the associations found in cohort 1, we enrolled another 206 OS cases and 360 normal controls in cohort 2, and we also tested all 5 APE1 polymorphisms. The analysis did not yield a significant deviation from HWE for all 5 APE1 polymorphisms in the control group (All *P* > 0.10). As in cohort 1, individuals carrying TG genotype and GG had significantly lower risk of developing OS than those with the WT genotype TT at rs1760944 (OR = 0.68, 95%CI 0.47–0.99; OR = 0.53, 95%CI 0.32–0.88, respectively). Individuals with G allele at rs1760944 had an almost 30% lower risk of developing OS than those with the T allele (OR = 0.71, 95%CI 0.56–0.92). Likewise, the combined study also showed the rs1760944 mutation to be significantly closely associated with risk of OS, and individuals with GG genotype and G allele had a significantly lower risk of developing OS (OR = 0.50, 95%CI 0.34–0.74; OR = 0.69, 95%CI 0.57–0.83, respectively).

### Association of rs1760944, with clinicopathological characteristics in OS patients

The association of rs1760944 with clinicopathological features in OS patients was assessed further. The results of stratification analysis with parameters of age at diagnosis, gender, tumor location, tumor size, tumor differentiation grade, metastasis at diagnosis, and response to chemotherapy are given in Table 3. OS patients carrying GG genotype at rs1760944 had a significantly decreased risk of low differentiation tumor (OR = 0.50, 95%CI 0.26–0.96). The results also showed that OS patients with allele G at rs1760944 had a much lower risk of low differentiation tumor (OR = 0.73, 95%CI 0.54–0.98). However, a positive association of rs1760944 with metastasis was also observed in OS patients (OR = 0.31, 95%CI 0.11–0.82). At the allele level, OS patients with G allele at rs1760944 were less susceptible to metastasis (OR = 0.63, 95%CI 0.43–0.92). However, no significant association of rs1760944 with other clinicopathological characteristics was observed.

**Table 3:** The association of rs1760944 with clinicopathological characteristics in OS patients.

Characteristic	N	Genotypes			Allele		
		TT	TG	GG	T		G
Age (Years)							
≤20	213	91	93	29		275	151
>20	165	72	70	23		214	116
OR (95% CI)		Reference	0.95 (0.61–1.47)	1.01 (0.54–1.88)		Reference	0.98 (0.73–1.33)
P value		—	0.46	0.56		—	0.49
Gender (n, %)							
Male	213	94	92	27		280	146
Female	165	69	71	25		209	121
OR (95% CI)		Reference	1.05 (0.68–1.63)	1.26 (0.67–2.36)		Reference	1.11 (0.82–1.50)
P value		—	0.46	0.29		—	0.27
Tumor Location							
Femur/Tibia/Fibula	307	130	134	43		394	220
Other	71	33	29	9		95	47
OR (95% CI)		Reference	0.85 (0.49–1.48)	0.82 (0.37–1.86)		Reference	0.89 (0.60–1.30)
P value		—	0.34	0.40		—	0.30
Tumor Size (cm)							
<6	194	86	83	25		255	133
≥6	184	77	80	27		234	134
OR (95% CI)		Reference	1.08 (0.70–1.66)	1.21 (0.65–2.25)		Reference	1.10 (0.82–1.48)
P value		—	0.41	0.33		—	0.29
Pathological Grade							
High Differentiation	193	76	84	33		236	150
Low Differentiation	185	87	79	19		253	117
OR (95% CI)		Reference	0.82 (0.53–1.27)	**0.50 (0.26–0.96)**		Reference	**0.73 (0.54–0.98)**
P value		—	0.22	0.004		—	0.04
Metastasis at Diagnosis							
Negative	296	121	128	47		370	222
Positive	82	42	35	5		119	45
OR (95% CI)		Reference	0.79 (0.47–1.32)	**0.31 (0.11–0.82)**		Reference	**0.63 (0.43–0.92)**
P value		—	0.21	0.01		—	0.01
Response to chemotherapy							
Poor	199	85	86	28		256	142
Good	179	78	77	24		233	125
OR (95% CI)		Reference	0.98 (0.63–1.51)	0.93 (0.50–1.75)		Reference	0.97 (0.72–1.30)
P value		—	0.25	0.01		—	0.04

### Association of rs1760944 with OS prognosis

**Figure 1:**
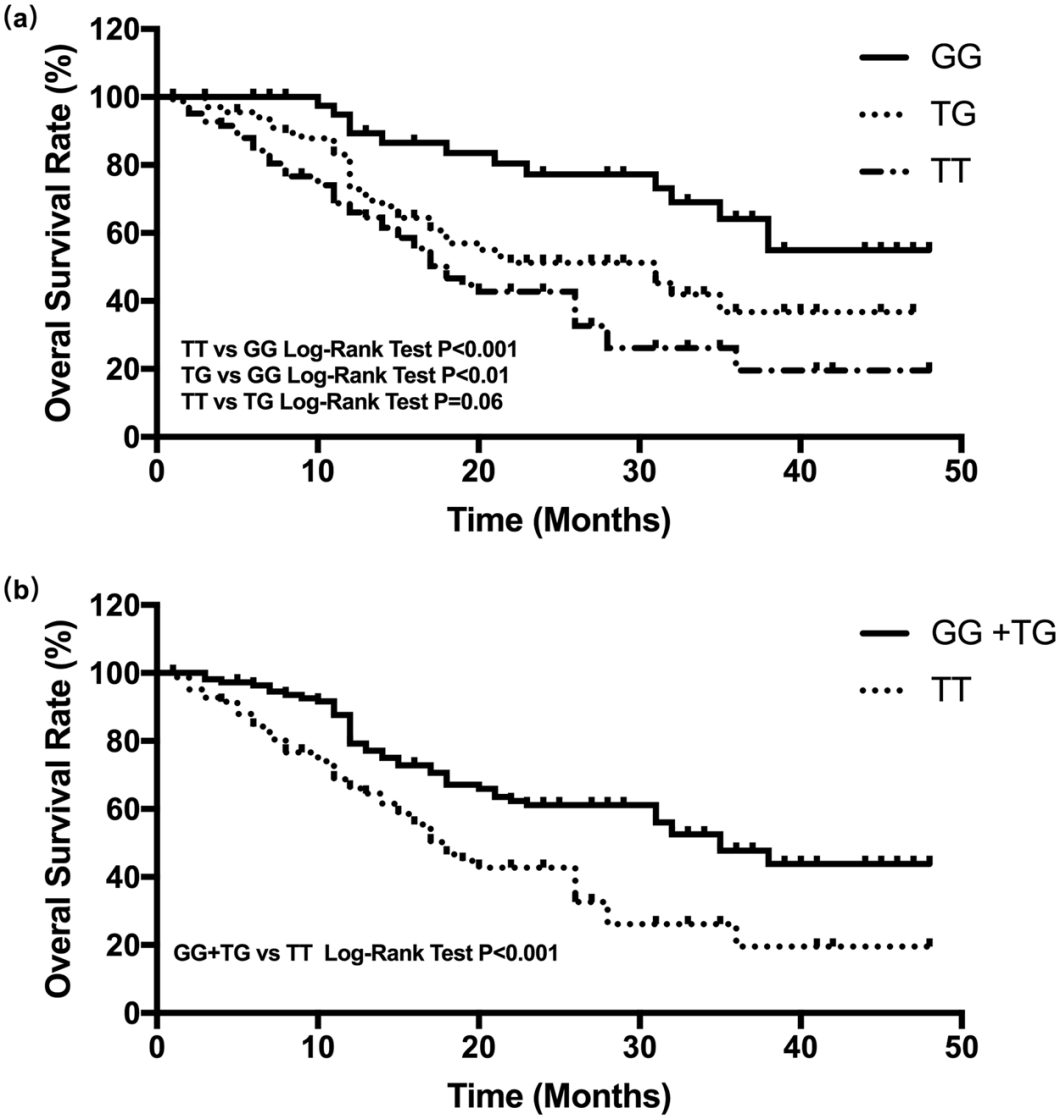
Kaplan-Meir survival curves; Kaplan-Meir survival curves of OS patients with different APE1 rs1760944 genotypes (**a**,**b**).

**Figure 2:**
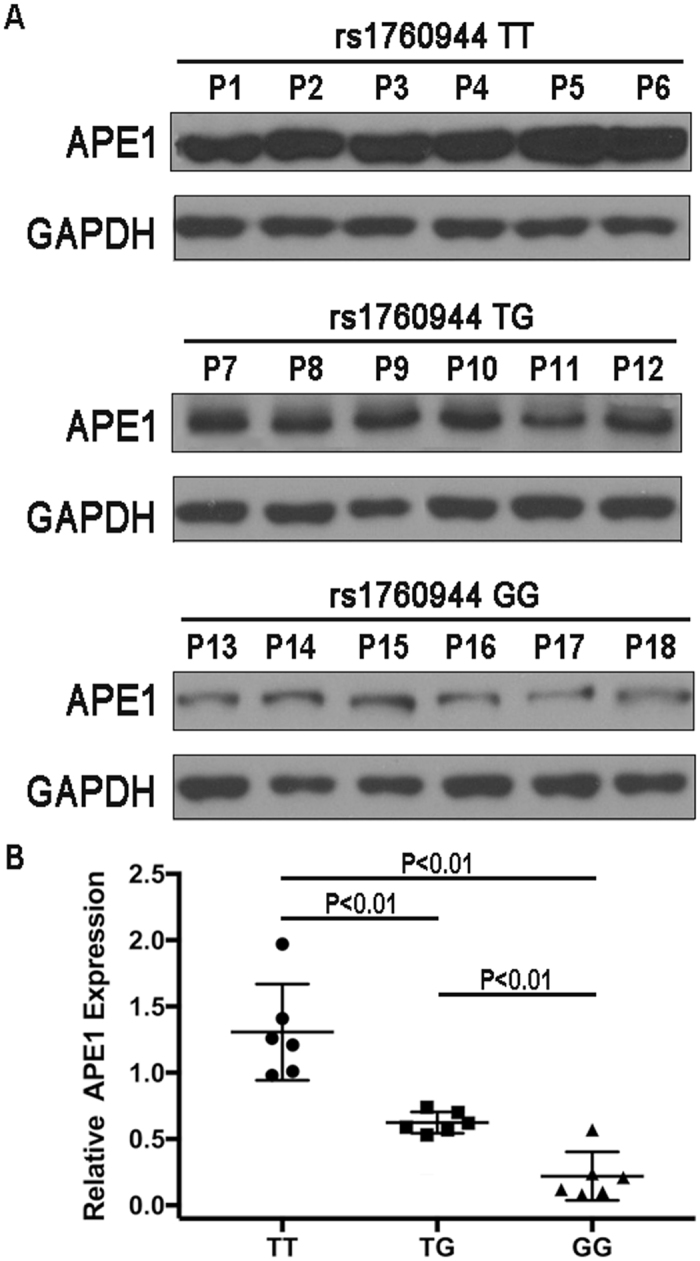
Association between rs1760944 polymorphism and APE1 expression. APE1 protein expression in OS tumor tissues from individuals with different rs1760944 genotypes was evaluated by western blotting; TT genotype (n = 6); TG genotype(n = 6); GG genotype (n = 6). (APE1, 34 kd; GAPDH, 37 kd).

Kaplan–Meier curves were constructed to evaluate the association of survival rate with rs1760944. Significant differences in overall survival were detected among OS patients with different genotypes at rs1760944 (Fig. 1). Kaplan-Meier curves and log-rank results demonstrated that OS patients carrying TG and GG at rs1760944 had longer survival time than those with TT genotype (Fig. 2A, *P* < 0.001, *P* < 0.01, respectively). Consistently, OS patients carrying G allele (GG + TG) at rs1760944 also had better survival (*P* < 0.001, Fig. 1b).

### Association of rs1760944 polymorphism with APE1 expression

To assess the effect of rs1760944 polymorphism on APE1 expression, protein expression levels of APE1 were further analyzed in OS patients. The effect of rs1760944 polymorphism on APE1 expression was evaluated using Western blotting. As shown in Fig. 2, the protein expression levels of OS patients carrying TG or GG genotype were significantly lower than those of individuals with the TT genotype. Taken together, the lower levels of APE1 expression in OS patients with G allele than those with other genotypes suggested that G allele at rs1760944 may be a protective genetic factor for OS.

## Discussion

APE1 is a ubiquitous multifunctional protein that has both DNA repair and redox regulatory activity. Although it was first identified as a DNA repair enzyme, accumulating evidence supports a role for APE1 in tumor development. In the present study, the association between rs1130409, rs1760944, rs1760941, rs2275008, and rs17111750 polymorphisms at APE1 on OS risk and prognosis were assessed in a 2-stage case-control study. These results suggested that G allele and GG genotype at rs1760944 could decrease the risk of development of OS and protected against low differentiation tumor and positive metastasis relative to the homozygous TT genotype. OS patients carrying TG and GG at rs1760944 had better survival than those with genotype TT. In addition, these results also showed that APE1 protein was underexpressed in individuals who carried the G allele. Taken together, these data suggested that the rs1760944 polymorphism could affect the susceptibility to OS, potentially by modulating APE1 protein expression.

It has been demonstrated that APE1 plays a critical role in many biological processes, such as cell proliferation and growth, cell cycle control, apoptosis, and angiogenesis, which are closely related to cancer development. Despite the complexity and limited knowledge of the roles played by APE1/in tumorigenesis, accumulating evidence suggested that APE1 has a considerable effect on cancer progression^18^. One previous study reported that the knockdown of APE1 could slow cell cycle progression and suppress tumor growth of ovarian cancer^19^. Inhibition of APE1 function was found to block the growth of tumor cell lines and significantly prevent endothelial cell proliferation and capillary formation, suggesting its potential promotive effect on tumor angiogenesis^20^. Similarly, inhibition of APE1 activity could impede pancreatic cancer cell growth and migration^21^. In osteosarcoma, Wang *et al*. reported that APE1 was overexpressed in osteosarcoma and a decrease in APE1 expression by siRNA was found to enhance cell sensitization to DNA damaging agents^22^. Similar to the results of the present work, Wang’s data also showed there to be a significant correlation between high levels of APE1 expression and reduced survival time. Jiang’s study indicated that APE1 could regulate angiogenesis in osteosarcoma by controlling TGFb pathway and decreasing phosphorylation of Smad3^23^. However, a previous study showed that knockdown of APE1 could markedly inhibit tumor angiogenesis by downregulating FGF2/FGFR3 in human osteosarcoma model^24^.

There are several polymorphisms in the APE1 gene, of which rs1130409 is the most widely studied. It has been reported to be associated with many cancers. However, the results of those studies remain controversial. Zhang *et al*. observed a significant association between rs1130409 polymorphism and susceptibility to ovarian cancer and that the TG/GG genotype and G allele were associated with a decreased risk of ovarian cancer^12^. Yuan *et al*. reported that this polymorphism may not play a major role in head and neck cancer (HNC) in Chinese individuals^25^. Similar results were also observed in gastric cancer^26^. A meta-analysis that covered 7 studies also showed no positive results between rs1130409 polymorphism and risk of prostate cancer risk in individuals of Asian descent^13^. Another pooled meta-analysis showed that this polymorphism had no effect on overall cancer risk, but stratification analysis indicated a significantly decreased risk of lung cancer in Asians^27^. However, Cao found the GG genotype at rs1130409 to have a significantly higher risk of renal cell carcinoma in Chinese individuals than rs1760944 does^28^. In breast cancer, neither rs1760944 nor rs1130409 were not found to be positively associated with the risk of breast cancer^29^. As in these previous works, no association was found between rs1130409 polymorphism and risk of OS risk in the Chinese population studied here. Some other researchers have found lung cancer patients bearing G allele at rs1130409 to have a significantly higher risk of radiation-induced pneumonitis than those with the wild TT genotype^30^.

rs1760944 was the other APE1 polymorphism that was most widely investigated in the tumor population. The association of rs1760944 with lung cancer susceptibility was first reported by Lu, who found a T-to G variant at rs1760944 in the promoter region associated with decreased risk of lung cancer^31^. Individuals with the homozygous GG genotype exhibited 46% lower risk of glioblastoma than the TT homozygote^32^. No significant association of rs1760944 with gastric cancer was observed among Koreans, but the investigator found gastric cancer patients bearing GT/GG genotypes to have a higher survival rate than those carrying TT in Chinese populations^26, 33^. A pooled meta-analysis supported the conclusion that rs1760944 polymorphism has a possible protective effect on cancer susceptibility among Asians^34^. Consistent with these data, these results also demonstrated that the homozygous GG genotype and heterozygote TG were associated with a lower risk of OS than the TT genotype. Lu also discovered that G allele could decrease APE1 mRNA expression levels by impairing the binding affinity of octamer-binding transcription factor-1 (Oct-1)^31^. In the present study, the level of APE1 expression among OS patients with different genotypes at rs1760944 was measured and results showed OS patients carrying G allele (TG + GG) to have higher levels of APE1 expression than those with the TT genotype. Although the mechanism underlying the association of rs1760944 with OS risk has been not identified, it is here speculated that this polymorphism may affect susceptibility to OS through the mechanism given above.

There have been relatively few reports of the association between rs2275008 and the risk of cancer. Kazma’s group investigated the role of several DNA repair gene polymorphisms with lung cancer^35^. As in the current work, they did not observe a positive association between rs2275008 polymorphism and the risk of lung cancer. Corral *et al*. have conducted a study to examine the associations of 182 haplotype tagging SNPs in 14 BER genes with colorectal adenoma risk, and rs17111750 was found to be closely associated with the risk of colorectal adenoma among African-Americans but not Asian-Pacific islanders^36^. This discrepancy may be attributed to differences among ethnicities. In the current study, no positive association was observed between rs17111750 and the risk of OS in Chinese individuals. The role of this polymorphism in other ethnic populations must also be investigated in future works. Until now, there have been few reports concerning the role of rs1760941 in tumor patients. The current study was the first investigation to analyze the association of rs1760941 with the risk of cancer. However, the data collected here indicate no statistically significant association between rs1760941 and the risk of OS.

In the present study, a case-control study was conducted in 2 cohorts. It is advantageous to perform replications of experiments in independent cohorts, which improves accuracy and reliability and is essential to biomarker studies. Obviously, there were several limitations. First, the sample size of the present study was relatively small. Especially, the specific role of rs2275008, rs17111750, and rs1760941 in the development of OS must be confirmed in a large cohort study due to the low frequency of mutations among Chinese individuals. Secondly, although APE1 expression has been found to be increased in OS patients carrying the GG genotype at rs1760944, but the specific mechanism was not clear. Lu’s results suggest that this polymorphism could affect the binding affinity of transcriptional factor (Oct-1)^31^. This study was conducted exclusively on Chinese Han participants, and the frequency of these 5 polymorphisms in other ethnic groups must be confirmed. More comprehensive studies involving larger independent cohorts of different ethnicities are warranted to validate these findings.

In conclusion, the current genetic assessment is the first study to evaluate the association of rs1130409, rs1760944, rs1760941, rs2275008, and rs17111750 with OS. The results suggested APE1 rs1760944 polymorphism is associated with decreased risk of developing OS and better survival of OS patients.

## Methods

### Participants

A 2-stage case-control study was conducted. During the first stage, 172 patients with primary OS treated in at department of orthopedics of Shandong Qianfoshan Hospital (Jinan, China) and 256 normal subjects treated between July 2008 and December 2012 were enrolled. The second stage included another independent set of 206 OS patients and 360 healthy individuals. All of these subjects were of Chinese Han descent. The diagnosis of OS was based on clinical and histological examination of resected specimens from OS patients. None of the OS patients had any history of other cancers and had undergone no prior treatment, including chemotherapy and radiotherapy, when first diagnosed with primary OS. All age- and gender-matched controls, who were unrelated to OS patients, were recruited and had no orthopedic disease or cancer. This study was approved by the Ethics Committee of Shandong Qianfoshan Hospital and written informed consent was obtained from all participants. All procedures were carried out in accordance with the principles of the Declaration of Helsinki.

### Blood samples and medical data extraction

Approximately 5 ml peripheral vein blood was collected into a tube containing EDTA from each recruited subject. Clinical and laboratory information regarding all OS patients were obtained from medical records, including age at the time of diagnosis, gender, tumor location, tumor size, tumor pathological grade, metastasis at diagnosis, and response to chemotherapy. Among all OS patients, 195 were enrolled in the follow-up study. Survival time was defined as the time from surgery to the date of OS-related death or last follow-up.

### SNP selecting and genotyping analysis

SNPs within the *APE1* gene were selected based on the criteria as following: 1. Previous studies have reported the possible association of the relevant SNP with cancer. 2. The minor allele frequencies (MAF) were greater than 0.05 in Chinese Han population. Therefore, we selected 5 SNPs as candidate SNPs, specifically rs1130409, rs1760944, rs1760941, rs2275008, and rs17111750 for APE1. In the present study, TaqMan SNP genotyping assays were used to confirm genotypes for these 5 SNPs within APE1 gene. Approximately 5 ml peripheral blood was collected from all subjects into an EDTA tube. Genomic DNA was isolated using a QIAamp DNA Blood Mini Kit (Qiagen, CA, U.S.) based on the manufacturer’s instructions and kept at −20 °C until use. Genotyping analysis was performed using custom TaqMan SNP genotyping assays (ThermoFisher, OK, U.S.). Genotyping and allele analysis were conducted with a TaqMan genotyping master mix (ThermoFisher) and an ABI Prism 7900HT genetic detection system according to the manufacturer’s instructions in a final volume of 25 µL including 12.50 µL master mix, 1.25 µL of assay Mix, 11.25 µL of ddH20. PCR conditions were set as follows: 95 °C for 15 s, 60 °C for 1 min, for 40 cycles and then genotyping or allele calling was performed with SDS 2.3 software. Genotype or allelic frequency was assessed based on allelic discrimination plots using automatic allele analysis. To ensure that the polymorphisms observed here were not due to technical variation, we randomly selected 10% of total subjects for repeated assays, and no inconsistent results were found.

### Protein isolation and Western blot analysis

According to the distribution of the rs1760944 genotype, all 378 OS patients were divided into 3 groups. To analyze the effect of the rs1760944 polymorphism on APE1 protein expression, 8 OS tumor tissues were randomly selected from each group and protein was isolated. Frozen tumor tissues were lysed with RIPA buffer containing protease inhibitor (Complete, Sigma, CA, U.S.) and PMSF, then homogenized on ice with a glass homogenizer. Lysates were sonicated and centrifuged at 12,000 rpm at 4 °C for 5 min to remove cell debris and supernatants were collected. A BCA assay kit (Beyotime Biotech, Shanghai, China) was used to measure protein concentration. Approximately, 30 µg of extracted protein was subjected to 10% SDS-PAGE and transferred to PVDF membranes (Millipore, MA, U.S.). Membranes were blocked with 5% non-fat milk in TBST buffer (containing 0.1% Tween-20), and then incubated with human APE1 antibody (1:1,000 dilution, #4128, Rabbit IgG, CST, U.S.) and GAPDH antibody (1:1,000 dilution, sc-32233; Mouse IgG, Santa Cruz, CA, U.S.) at 4 °C overnight. Horseradish peroxidase (HRP)-conjugated anti-rabbit IgG and anti-mouse IgG were used as secondary antibody (1:5,000 dilution, Beyotime Biotech, Shanghai, China). Signals were captured using a CCD camera system (Bio-Rad) with an HRP chemiluminescent kit (Beyotime Biotech, Shanghai, China). APE1 protein expression was normalized to GAPDH by calculating the relative expression.

### Statistical analysis

Categorical data from OS patients and normal controls were compared using a Chi-square test. An association between APE1 genotypes and the risk of OS was estimated with odds ratios (ORs) using an unconditional logistic regression model. Survival probabilities were estimated using a Kaplan–Meier analysis, and significant differences were analyzed using a log-rank test. Deviations from Hardy-Weinberg equilibrium were assessed using a Chi-square test. Differences in APE1 expression among genotypes were compared with the Student’s t-test. All statistical analyses were performed with SPSS 22.0 (SPSS Inc., Chicago, IL, U.S.) and GraphPad Prism 6.0 (GraphPad, CA, U.S.). *P* < 0.05 was considered statistically significant.
